# Endovascular treatment of the cavernous sinus dural arteriovenous fistula: current status and considerations

**DOI:** 10.7150/ijms.45210

**Published:** 2020-05-01

**Authors:** Kun Hou, Guichen Li, Tengfei Luan, Kan Xu, Jinlu Yu

**Affiliations:** 1 Department of Neurosurgery, The First Hospital of Jilin University, Changchun, 130021, China; 2 Department of Neurology, The First Hospital of Jilin University, Changchun, 130021, China

**Keywords:** cavernous sinus, dural arteriovenous fistula, endovascular treatment, review

## Abstract

A cavernous sinus dural arteriovenous fistula (CS-DAVF) is an abnormal arteriovenous communication involving the dura mater within or near the CS wall. The dural arteries from the internal carotid artery and external carotid artery supply the CS-DAVF, and the superior ophthalmic vein (SOV) and inferior petrous sinus (IPS) are frequent venous drainers. In CS-DAVF cases, high-risk lesions require treatment. Endovascular treatment (EVT) has been the first-line option for CS-DAVFs. To our knowledge, a review of the EVT of CS-DAVFs is lacking. Therefore, in this paper, we review the available literature on this issue. In addition, some illustrative cases are also provided to more concisely expound the EVT of CS-DAVFs. According to the recent literature, transvenous embolization via the IPS is considered the most effective method for EVT of CS-DAVFs. In addition, the transorbital approach is another reasonable choice. Other venous approaches can also be tried. Because of the low cure rate, transarterial embolization for CS-DAVFs is limited to only highly selected patients. In the EVT of CS-DAVFs, various agents have been used, including coil, Onyx, and n-butyl cyanoacrylate, with coil being the preferred one. In addition, when EVT cannot obliterate the CS-DAVF, stereotactic radiotherapy may be considered. In general, despite various complications, EVT is a feasible and effective method to manage CS-DAVFs by way of various access routes and can yield a good prognosis.

## Introduction

Cavernous sinus dural arteriovenous fistula (CS-DAVF) is the abnormal communication between the CS and the dural arteries from the internal carotid artery (ICA) and external carotid artery (ECA), commonly caused by CS recanalization after thrombosis 1.

In CS-DAVFs, retrograde arterialized flow in the draining veins results in characteristic symptoms 2. Low-flow asymptomatic CS-DAVFs can close spontaneously, but high-flow symptomatic CS-DAVFs can rarely disappear spontaneously 3. Therefore, treatment is recommended for symptomatic CS-DAVFs. Currently, endovascular treatment (EVT) has become the first-line choice for CS-DAVFs 4. In addition, adjuvant radiosurgery may be considered after unsatisfactory EVT 5.

Currently, a comprehensive review of EVT for CS-DAVFs is still lacking. In this paper, we review the available literature on this issue. In addition, some illustrative cases are also provided to more concisely expound the EVT of CS-DAVFs.

## Critical vessels in the CS region

The CS is an extradural space located in the bilateral parasellar compartment. The ICA passes through the CS and gives rise to several small branches supplying the dura. In addition, the ECA also supplies the CS through its dural branches 6, 7. The blood supply of the CS can be bilateral 8. There are constant anastomoses between the ICA and ECA, which are called “dangerous anastomoses” 9.

CS is not a single venous lake but a venous plexus. It receives venous drainage from the superior and inferior ophthalmic veins (OVs) anteriorly and from the sphenoparietal sinus and the superficial middle cerebral vein laterally, and drains posteriorly to the superior and inferior petrous sinuses and basilar plexus, inferiorly to the pterygoid plexus 10, 11. The bilateral CSs communicate with each other through the intercavernous sinus 8, 12.

**Figure 1:**
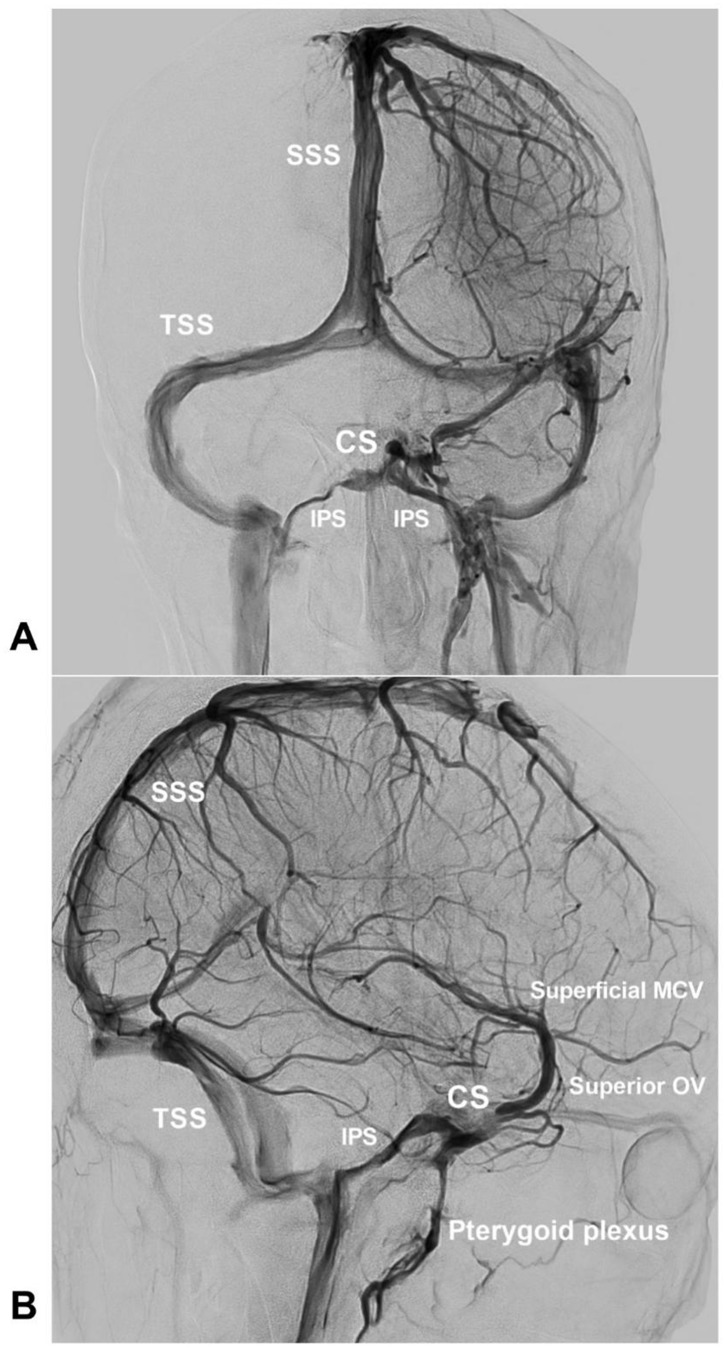
** Normal venous drainage of the CS.** An angiogram of the left ICA of the venous phase in AP (A) and lateral (B) views shows the CS and the venous structures around the CS. **Abbreviations:** AP, anteroposterior; CS, cavernous sinus; ICA, internal carotid artery; IPS, inferior petrous sinus; MCV, middle cerebral vein; OV, ophthalmic vein; SSS, superior sagittal sinus; TSS: transverse sinus.

The normal venous drainage of CS is shown in Figure 1.

## Angioarchitecture and classification of CS-DAVF

### Feeding artery

Feeders of CS-DAVFs are from abundant ECA and/or ICA branches, in which the middle meningeal artery (MMA), accessory meningeal artery, and ascending pharyngeal artery (APhA) are the most clinically significant branches 13-16. These feeders may be bilateral in nearly 70% of cases 16, 17. Under normal circumstances, these small dural branches may not be visible on catheter angiography but become hypertrophic or enlarged when supplying CS-DAVFs 18.

### Fistula point

The CS has several compartments, but usually, the fistulous portion does not involve the entire CS; the fistulous portion is also called the shunted pouch (SP) 19-22. The SP is a tubular or elliptical structure that is separated from the main sinus lumen and the site where multiple feeding arteries converge and continue to the CS 23.

The SP is mainly situated at the posterior compartment of the CS and connects with the intercavernous sinus. It might have one or more of the following angiographic findings: a) an early opacified area in the early arterial phase of catheter angiography, b) a jellyfish-like sign denoting the branches of arterial supply converging at the fistula point, c) changes in the density of the contrast medium from dark in the artery to gray in the veins on the fistula point, d) enlarged and tortuous arterial feeders that can directly point to the fistula point 24, 25.

The SP may be single or multiple, and it can be divided into dural, extradural, or osseous types based on the converging site of arterial feeders 17, 26.

### Venous drainage

In the high pressure gradient environment of CS-DAVFs, the arterialized veins reverse the direction of their normal inlet, and the drainers are highly variable 16. Superior ophthalmic vein (SOV) drainage is a frequent finding that can be present in 88% of patients with CS-DAVFs, while the rate is only 42% with inferior petrous sinus (IPS) drainage and 34% with cortical venous drainage 27.

The contralateral or bilateral drainage can also be visualized 13, 16. Rarely, venous reflux from CS can drain into the posterior fossa and brainstem by bridging veins 28. The draining vein can be stenotic or obliterated due to thrombus formation 27, 29-31.

### Classification

The Borden and Cognard grades are most commonly used for the classification of DAVFs. Their grading criteria are based on the venous drainage pattern, which is also applicable to CS-DAVFs 32-34. However, the CS-DAVF has its own anatomical and angiographic characteristics and should be classified more specifically 35-37. The following classification systems are helpful to guide the EVT of CS-DAVFs 38-41.

In 2005, Suh et al. divided the CS-DAVF into 3 types: proliferative CS-DAVF receiving numerous arterial feeders with rapid filling of the CS and drained by multiple venous outlets, late restrictive CS-DAVF draining primarily into the superior and inferior OVs, and restrictive CS-DAVF, which is the transitional type between the proliferative and late restrictive CS-DAVFs 38.

In 2015, Thomas et al. divided CS-DAVFs into 5 types, with types 1-4 designed for DAVFs 39. Type 1 has posterior/inferior drainage (IPS, superior petrous sinus (SPS), pterygoid plexus). Type 2 has posterior/inferior and anterior drainage (superior and inferior OVs). Type 3 has anterior drainage only. Type 4 has retrograde drainage into cortical veins.

In 2017, Wenderot et al. divided the CS-DAVF into 3 types: type 1 has free IPS outflow from the affected CS; type 2 has bilateral CS drainage but unilateral IPS occlusion, and type 3 has no IPS outflow at all 40.

These classification systems provide useful information on fistula anatomy and hemodynamics, which could further aid in the planning of fistula access during EVT 42.

## Outline of EVT

Currently, various EVTs are available for CS- DAVFs, mainly including transfemoral transvenous embolization (TVE) and transarterial embolization (TAE), a transorbital puncture approach, and direct puncture or surgical exposure to obtain transvenous access to the CS 3, 5, 43.

In CS-DAVFs, TAE is usually difficult because most CS-DAVFs are fed by numerous and tiny dural branches from the ICA and ECA. In addition, TAE has a low cure rate. Therefore, TAE should be used only in highly selected cases 13, 44.

Nevertheless, TVE has become the mainstream EVT for CS-DAVFs, of which the transfemoral IPS approach is the favorite, shortest, and most direct route to the CS 1, 15. In TVE, precise identification of the SP location is crucial because targeted dense embolization of the SP can reduce the amounts of embolic agents used and thus avoid complications 24, 25, 45.

If the transfemoral IPS approach is inaccessible, an alternative transorbital approach can be tried, which includes direct puncture of the superior and inferior OVs and CS as well as puncture of the superior OV after surgical exposure 5.

In the EVT of CS-DAVFs, various materials have been used, including coil, Onyx (Medtronic, Irvine, California, USA), and n-butyl cyanoacrylate (NBCA) 46. For TVE, coil is the preferred material, and Onyx can also be used in combination or alone 3. For TAE, NBCA is an effective agent due to its good ability in distal penetration 13. However, Onyx is also a promising choice due to its nonadhesive nature, allowing for a longer injection time 47.

## TVE

### Transfemoral venous approach

For the CS-DAVF with a visible IPS, trans-IPS access was successful in nearly 90% of the cases 30, 48. The contralateral IPS can also serve as an alternative route 15, 49. 3D rotational venography of the internal jugular vein (IJV) is very useful for revealing the details of the IPS and creating a plan to pass it 43, 50. In addition, preoperative CT angiography (CTA) can also help to localize the IPS 51.

To perform TVE, the reopening of an occlusive IPS is feasible, and the success rate ranges from 50% to 90% 15, 16. The major factors affecting the reopening of an occlusive IPS are the duration and length of occlusion. It is difficult to reopen an IPS with a long duration and length of occlusion 16, 43. During reopening the IPS, a standard hydrophilic-polymer- jacketed 0.035-inch guidewire can advance as a frontier wire for probing the IPS. Then, the trace of the guidewire on the roadmap image can serve as a guide for microcatheter navigation through the IPS 27, 50. In addition, the microwire looping technique for breaching the occluded IPS under the reinforcement of guiding system support is also helpful, during which the 5F catheter fixed to the outlet of IPS provides a stable support 29, 51, 52.

In addition to targeted dense SP packing, the following details should also be considered: 1) the origin of the superior and inferior OVs should be packed to ensure that the fistula is not redirected into the orbit, and 2) cortical and brainstem venous drainage should be occluded with priority 27,29,30, 38,53.

**Figure 2:**
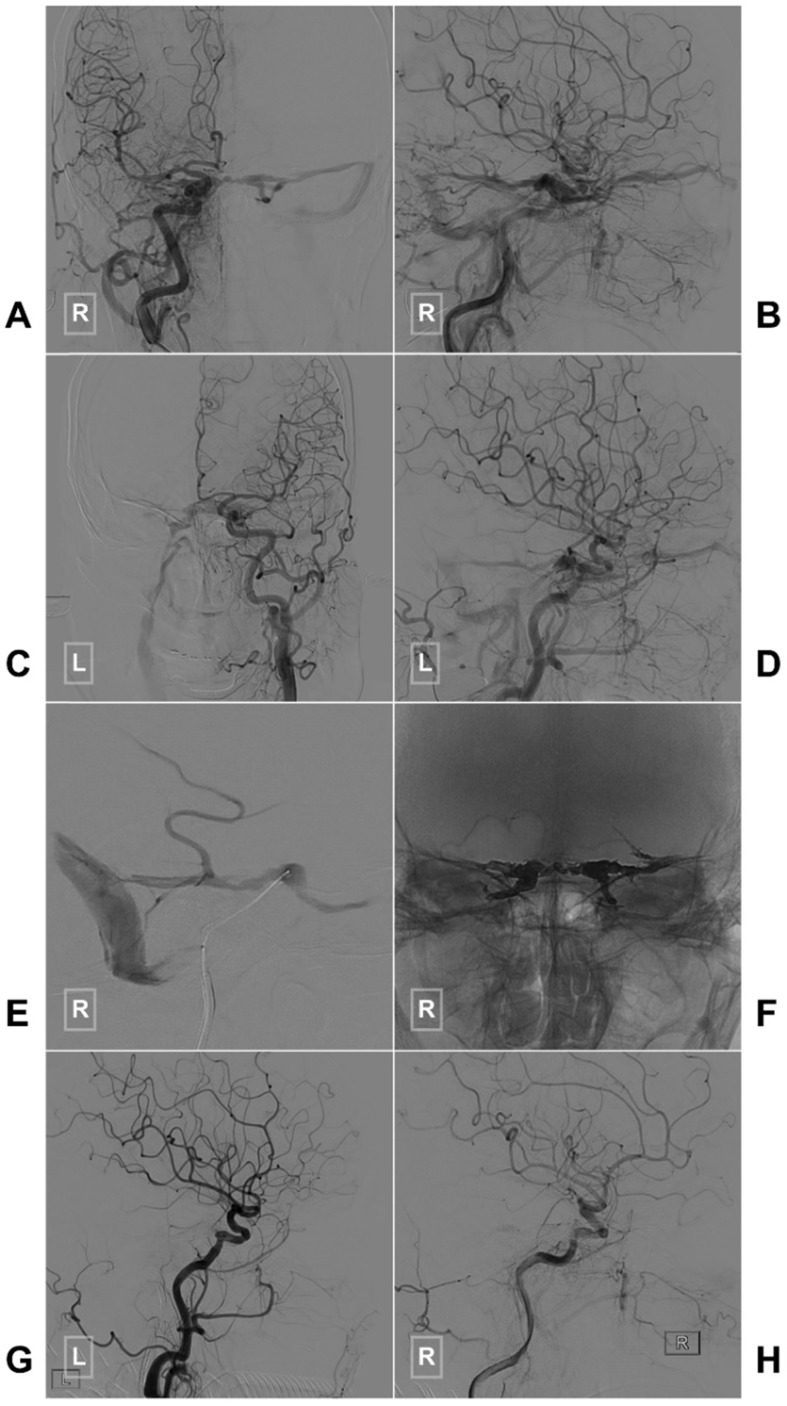
** Typical case of a CS-DAVF with TVE via the IPS.** An angiogram of the right (A-B) and left (C-D) common carotid arteries in AP (A, C) and lateral (B, D) views shows a CS-DAVF supplied by the bilateral dural branches of ECA and ICA. E, The microcatheter advances to the CS via the right IPS. F, The bilateral CSs are packed by Onyx. G-H, A postprocedural angiogram of the right common carotid artery in the lateral view shows that the CS-DAVF is obliterated. **Abbreviations:** AP, anteroposterior; CS, cavernous sinus; CS-DAVF, cavernous sinus dural arteriovenous fistula; ECA, external carotid artery; ICA, internal carotid artery; IPS, inferior petrous sinus; L: left; R: right; TVE, transvenous embolization.

If the SP packing is not satisfactory or the CS-DAVF belongs to the proliferative type according to Suh et al.'s classification, the entire CS has to be packed densely 38. A case of CS-DAVF with entire CS packing via IPS is illustrated in Figure 2.

In addition to the trans-IPS approach, other transfemoral routes can also be used to access the CS, e.g., the transpterygoid plexus approach 48, 54-57. However, these routes can only be used after unsuccessful trans-IPS attempts 11, 56.

### Transorbital approach

Transorbital access to the CS belongs to the transvenous approach, including direct puncture of the superior and inferior OVs and CS or direct puncture of the SOV after surgical exposure 5, 15. The superior and inferior OVs can be punctured under sonographic and biplane roadmap guidance 58, 59. After anterior orbitotomy, the exposed SOV can be punctured easily, with the advantage of a lower possibility of injuring the intraorbital structure and reduced distance to the CS 5, 60. When direct puncture or orbitotomy to access the SOV fails, a more radical method of performing orbital removal for catheterization of the SOV is another practical alternative 61.

Previously, transorbital CS puncture was not widely adopted due to the lack of direct or indirect visualization. Currently, the modern angiographic system has advanced greatly with the progression of software dedicated to reconstructing cone-beam computed tomography (CT). Currently, direct puncture of the confluence of the superior and inferior OVs under the guidance of planning software is safe and feasible 59.

### Other transvenous approaches

#### Puncture or incision access through other head and neck veins

In cases with the SOV as the main drainage vessel, access to the CS via the facial vein (FV) is possible 11, 62. Matsumoto et al. (2017) reviewed previous reports of the transfemoral FV approach to the CS, and the success rate varied from 50%-100% 11. In cases of unsuccessful transfemoral FV, direct puncture or incision of the FV can be attempted 62-64.

In addition to the FV, other veins, such as the superficial temporal vein, retromandibular vein, and jugular vein, can also be punctured to access the CS 11, 65. To more efficiently puncture these veins, a small skin incision can be made 66. It is helpful to create a microwire loop, which can increase the possibility of passing these abruptly angulated and tortuous veins 11, 56, 67.

#### Transcranial vein access

Rarely, transcranial puncture to the superficial middle cerebral vein can be used to perform EVT for CS-DAVF, which combines open surgery and endovascular techniques 68, 69. Especially when the CS-DAVF has caused an intracranial hematoma, it is appropriate to evacuate the hematoma and embolize the CS-DAVF in a single session 35, 70. In 2017, Akamatsu et al. reviewed four cases of CS-DAVFs, all of which underwent successful EVT via the superficial middle cerebral vein 70.

#### Transforamen ovale access

The approach to CS through the foramen ovale and catheterization of the skull base emissary vein can be selected as an alternative when there is no possibility of a conventional venous approach. For instance, Cabral De Andrade et al. (2012) and Gil et al. (2013) performed EVT of CS-DAVFs through the foramen ovale 71, 72.

## Transarterial embolization

When TVE is difficult or the DAVF only involves a single-side dura of the CS, it is limited in size, and with few dural feeders, TAE can be attempted 44. If the microcatheter tip can be wedged into the fistula with an ideal position, the TAE is safe and effective 22, 73. The MMA and accessory meningeal artery provide gold routes to occlude the CS-DAVF 74. In addition, in CS-DAVFs fed by APhA, TAE via APhA or combined with other routes can be a reasonable and effective choice 3, 14, 75.

During TAE, the Onyx and NBCA need to penetrate into the fistula and draining veins, as well as retrogradely into the branches of other arterial feeders 76. In early years, NBCA was more popular in TAE for CS-DAVFs 13. Currently, Onyx has become the first choice 44. During TAE, to avoid embolization of dangerous anastomoses, protective balloon navigation and inflation to the cavernous ICA during injection is a feasible method 32, 77.

**Figure 3:**
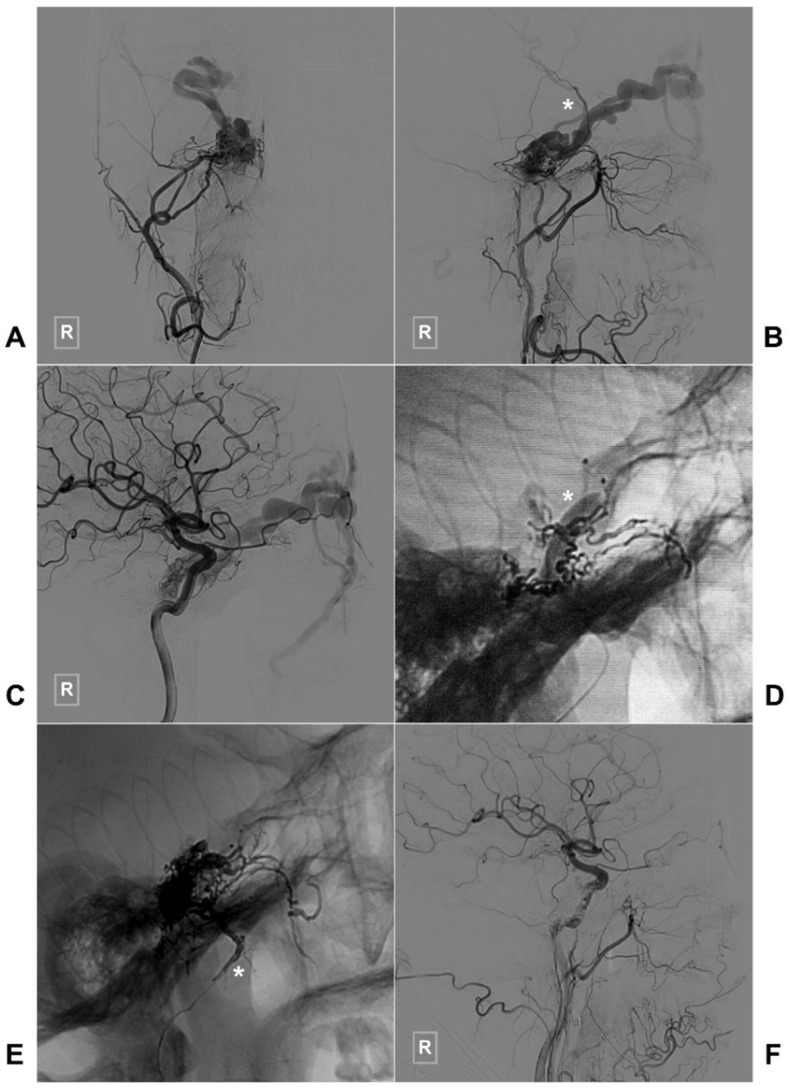
** Typical case of a CS-DAVF with TAE via the right MMA.** An angiogram of the right ECA in AP (A) and lateral (B) views shows that the CS-DAVF is supplied by the MMA and accessory meningeal artery and drains to the superior ophthalmic vein and superficial middle cerebral vein (asterisk in B). C, An angiogram of the right ICA in the AP view shows that the dural branches of the ICA also supply blood to the CS-DAVF. D, During TAE, a balloon (asterisk) in the ICA is inflated to protect the ICA. E, An unsubtracted angiogram shows the Onyx casting via MMA (asterisk), F: An angiogram of the right common carotid artery shows that the CS-DAVF is obliterated. **Abbreviations:** ECA: external carotid artery, CS: cavernous sinus, DAVF: dural arteriovenous fistula, DSA: digital subtraction angiography, ICA: internal carotid artery, MMA, middle meningeal artery TAE: transarterial embolization.

**Figure 4:**
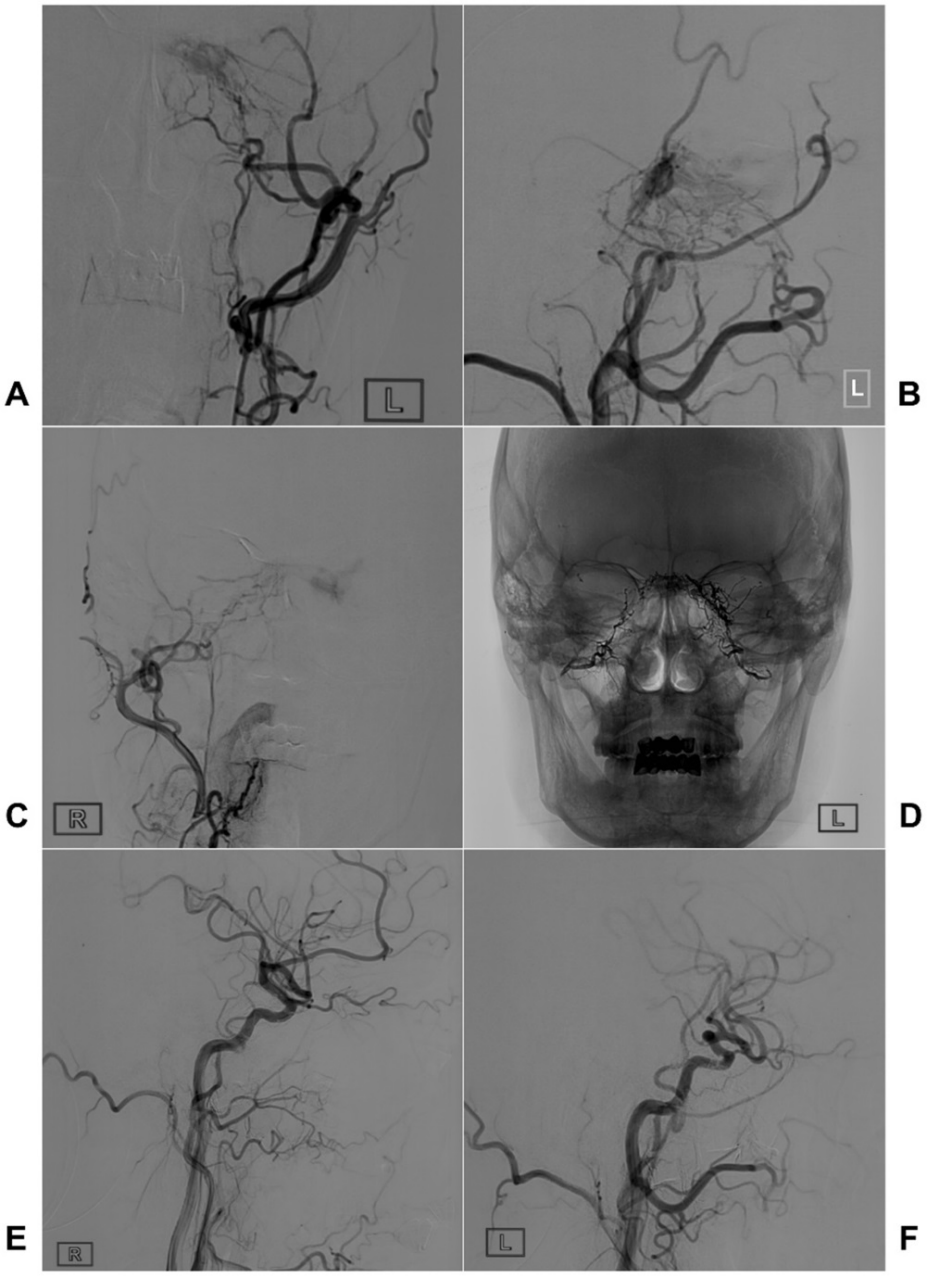
** Typical case of a CS-DAVF with TAE via bilateral MMAs.** An angiogram of the left ECA in AP (A) and lateral (B) views shows that the CS-DAVF is supplied by the MMA and internal maxillary artery. C, An angiogram of the right ECA in the AP view shows that the right MMA also supplies blood to the CS-DAVF. D, An unsubtracted angiogram shows the Onyx casting via bilateral MMAs. E-F: A follow-up angiogram of the right (E) and left (F) common carotid arteries six months later shows that the CS-DAVFs are completely obliterated. **Abbreviations:** AP, anteroposterior; CS, cavernous sinus; CS-DAVF, cavernous sinus dural arteriovenous fistula; ECA, external carotid artery; MMA, middle meningeal artery; TAE, transarterial embolization.

Two illustrative cases with TAE of CS-DAVFS are provided in Figures 3 and 4.

## Combined radiosurgery

Currently, stereotactic gamma-knife radiosurgery (SGKR) with minimal invasiveness has become an alternative primary management for DAVFs. In Hung et al.'s (2019) study, after SGKR, CS-DAVFs were more likely to be obliterated than non-CS-DAVFs 78. Therefore, when EVT cannot obliterate the CS-DAVFs, stereotactic SGKR may be considered 5.

Using a therapeutic radiation dose of 20-50 Gy, SGKR can induce injury to the targeted vessel, thus obliterating the DAVFs. Rates of up to 90% obliteration and 85% symptomatic improvement can be achieved 79, 80.

However, the drawbacks of SGKR are continued symptoms and risk of hemorrhage in the presence of retrograde cortical drainage or eye symptoms during the latency period 80. To overcome this limitation, a combined approach consisting of EVT and the following SGKR can be used 79.

## Prognosis and complications

In general, EVT, by way of various access routes, is a feasible and effective method to manage CS-DAVFs 27, 67, 81.

### Angiographic results and prognosis

The angiographic results of EVT can be classified into complete cure, minor residual shunt with occlusion of the superior and inferior OVs and CS, partial effect if shunt reduction was <80%, and failed attempt if there was residual filling of the superior and inferior OVs and deep venous system or cortical vein 27.

In EVT, the rate of total occlusion of CS-DAVFs varies from 51.7% to 90% using coils, from 80% to 100% using coils and NBCA, and from 76.9% to 100% using coils and Onyx 5, 13, 16, 41, 44, 48, 82, 83. A higher rate of complete occlusion is observed using liquid agents combined with coils than using coils alone.

However, complete occlusion is not the ultimate goal of EVT for CS-DAVFs 83. The primary goal should be eliminating cortical venous reflux and major retrograde shunt flow because long-term follow-up shows that the residual shunt flow can eventually disappear 83, 84. EVT of CS-DAVFs can result in a good prognosis.

### Complications

The overall rate of complications ranges from 2.3% to 53.3% 16, 41, 48, 83. In recent years, the rate has decreased significantly 16, 83. In de Castro-Afonso et al.'s (2018 and 2019) studies with more than 60 patients, the complication rate was 3.2% 83, 85. In Alexander et al.'s (2019) study with 267 patients, the complication rate was 3.6%, and permanent symptomatic complications only occurred in 0.51% of the patients 16.

Compared with TVE, TAE has a higher complication rate 16, 86. In TVE, coil embolization has fewer complications than liquid embolic agents 16, 87. The complications involved in EVT of CS- DAVFs can be divided into technical complications, cranial nerve palsies (CNPs), hemorrhagic and ischemic complications, and other complications 4.

#### Technical complications

In EVT, the microwire and microcatheter can lead to vessel injury 32. During TVE, navigation through an occlusive IPS may cause venous injury by the sharp microwire tip, and other transvenous routes have a higher rate of complications with possible venous rupture 67. In transorbital approaches, the complications include supraorbital nerve and cranial nerve injuries, injury to the eyeball, infection, bleeding from the superior and inferior OVs, and orbital or subarachnoid hemorrhage 56, 67. Therefore, careful preoperative planning, meticulous manipulation during puncture, and vigilant postoperative management are necessary to avoid these technical complications 88.

#### Cranial nerve palsy

In TAE, CNPs can result from compromise of the blood supply of relevant cranial nerves or inadvertent nontargeted embolization of dangerous ECA-ICA anastomoses 44, 81, 89. In TVE, CNPs may result from the mass effect of the overpacked CS or direct injury by coils or microwire/microcatheter 15, 30, 54, 55, 90.

In all CNPs, abducens nerve palsy is the most common after TVE and can occur immediately or delayed; usually, most CNPs recover within 6 months 41, 91. To avoid CNPs in TVE, the embolic material volume should be reduced, so superselective targeted occlusion of the SP is necessary 24, 45.

#### Other complications

In TAE, the most dangerous complication with liquid embolic agents is reflux through the meningohypophyseal or inferolateral trunks into the ICA and subsequent cerebral infarction, which must be avoided 16. Balloon protection in the ICA can be helpful.

In EVT, including TAE and TVE, due to direct toxicity from the dimethyl sulfoxide/Onyx combination, Onyx embolization of CS-DAVFs can carry the potential risk of trigeminocardiac reflex-induced bradycardia 27, 33, 92-95. Therefore, interventional neuroradiologists should bear in mind this rare consequence prior to injection of the embolic material 92.

Rarely, EVT for CS-DAVFs can result in venous brain stem infarction, which is suspected to be caused by insufficient packing of the posterior part of the CS, which diverts the embolic agent into the deep venous and brainstem venous system. Hence, during EVT for CS-DAVFs, occluding the entrance of bridging and cortical veins is very important 48, 84, 96.

## Summary

In summary, for higher risk CS-DAVFs, EVT is the first-line option. The transvenous approach via the IPS is considered the most effective method. The transorbital approach to obtain transvenous access to the CS is another choice. For CS-DAVFs, it is usually difficult to achieve a high cure rate with TAE. With regard to the embolic materials, coil, Onyx, and NBCA can be used. Coil is the preferred material. If the EVT cannot obliterate the CS-DAVFs, SGKR may be considered after obliteration of the risk factors. Despite various complications, EVT is an effective method to treat CS-DAVFs.

## References

[1] Hiramatsu M, Sugiu K, Hishikawa T, Nishihiro S, Kidani N, Takahashi Y (2019). Results of 1940 embolizations for dural arteriovenous fistulas: Japanese Registry of Neuroendovascular Therapy (JR-NET3). J Neurosurg.

[2] Zyck S, Gould GC (2019). Fistula, Dural Arteriovenous. StatPearls. Treasure Island (FL).

[3] Fang B, Qian C, Yu J, Xu L, Jiang D, Xu J (2018). Transarterial Embolization of Cavernous Sinus Dural Arteriovenous Fistulas with Ipsilateral Inferior Petrosal Sinus Occlusion via the Ascending Pharyngeal Artery. World Neurosurg.

[4] Henderson AD, Miller NR (2018). Carotid-cavernous fistula: current concepts in aetiology, investigation, and management. Eye (Lond).

[5] Phan K, Xu J, Leung V, Teng I, Sheik-Ali S, Maharaj M (2016). Orbital Approaches for Treatment of Carotid Cavernous Fistulas: A Systematic Review. World Neurosurg.

[6] Martins C, Yasuda A, Campero A, Ulm AJ, Tanriover N, Rhoton A Jr (2005). Microsurgical anatomy of the dural arteries. Neurosurgery.

[7] Seoane E, Rhoton AL Jr, de Oliveira E (1998). Microsurgical anatomy of the dural collar (carotid collar) and rings around the clinoid segment of the internal carotid artery. Neurosurgery.

[8] Rhoton AL Jr (2002). The cavernous sinus, the cavernous venous plexus, and the carotid collar. Neurosurgery.

[9] Salaud C, Decante C, Ploteau S, Hamel A (2019). Implication of the inferolateral trunk of the cavernous internal CAROTID artery in cranial nerve blood supply: Anatomical study and review of the literature. Ann Anat.

[10] Rhoton AL Jr (2002). The sellar region. Neurosurgery.

[11] Matsumoto A, Okauchi M, Shindo A, Kawanishi M, Tamiya T (2017). Cavernous sinus dural arteriovenous fistula treated by facial vein direct puncture: Case report and review of the literature. Interv Neuroradiol.

[12] Rhoton AL Jr, Harris FS, Renn WH (1977). Microsurgical anatomy of the sellar region and cavernous sinus. Clin Neurosurg.

[13] Liu HM, Huang YC, Wang YH, Tu YK (2000). Transarterial embolisation of complex cavernous sinus dural arteriovenous fistulae with low-concentration cyanoacrylate. Neuroradiology.

[14] Gross BA, Albuquerque FC, Moon K, McDougall CG (2017). The road less traveled: transarterial embolization of dural arteriovenous fistulas via the ascending pharyngeal artery. J Neurointerv Surg.

[15] Rhim JK, Cho YD, Park JJ, Jeon JP, Kang HS, Kim JE (2015). Endovascular Treatment of Cavernous Sinus Dural Arteriovenous Fistula With Ipsilateral Inferior Petrosal Sinus Occlusion: A Single-Center Experience. Neurosurgery.

[16] Alexander MD, Halbach VV, Hallam DK, Cooke DL, Ghodke BV, Dowd CF (2019). Long-Term Outcomes of Endovascular Treatment of Indirect Carotid Cavernous Fistulae: Superior Efficacy, Safety, and Durability of Transvenous Coiling Over Other Techniques. Neurosurgery.

[17] Rhim JK, Cho YD, Yoo DH, Kang HS, Cho WS, Kim JE (2018). Endovascular Treatment of Bilateral Cavernous Sinus Dural Arteriovenous Fistula: Therapeutic Strategy and Follow-Up Outcomes. Korean J Radiol.

[18] Choi BJ, Lee TH, Kim CW, Choi CH (2009). Reconstructive treatment using a stent graft for a dural arteriovenous fistula of the transverse sinus in the case of hypoplasia of the contralateral venous sinuses: technical case report. Neurosurgery.

[19] Kadooka K, Tanaka M, Sakata Y, Ideguchi M, Inaba M, Hadeishi H (2018). Efficacy of Cone Beam Computed Tomography in Treating Cavernous Sinus Dural Arteriovenous Fistula. World Neurosurg.

[20] Takahashi S, Sakuma I, Tomura N, Watarai J, Mizoi K (2004). Transvenous embolization of dural arteriovenous fistula of the cavernous sinus. Fistulous points and route of catheterization. Interv Neuroradiol.

[21] Kiyosue H, Tanoue S, Hori Y, Hongo N, Mori H (2015). Shunted pouches of cavernous sinus dural AVFs: evaluation by 3D rotational angiography. Neuroradiology.

[22] Xu K, Yang X, Li C, Yu J (2018). Current status of endovascular treatment for dural arteriovenous fistula of the transverse-sigmoid sinus: A literature review. Int J Med Sci.

[23] Kiyosue H, Tanoue S, Okahara M, Hori Y, Kashiwagi J, Sagara Y (2013). Angioarchitecture of transverse-sigmoid sinus dural arteriovenous fistulas: evaluation of shunted pouches by multiplanar reformatted images of rotational angiography. AJNR Am J Neuroradiol.

[24] Guo H, Yin Q, Liu P, Guan N, Huo X, Li Y (2018). Focus on the target: Angiographic features of the fistulous point and prognosis of transvenous embolization of cavernous sinus dural arteriovenous fistula. Interv Neuroradiol.

[25] Sato M, Izumi T, Matsubara N, Nishihori M, Miyachi S, Wakabayashi T (2018). Evaluation for shunted pouches of cavernous sinus dural arteriovenous fistula and the treatment outcome of transvenous embolization. Interv Neuroradiol.

[26] Kannath SK, Rajan JE, Sarma SP (2017). Anatomical localization of the cavernous sinus dural fistula by 3D rotational angiography with emphasis on clinical and therapeutic implications. J Neuroradiol.

[27] Kirsch M, Henkes H, Liebig T, Weber W, Esser J, Golik S (2006). Endovascular management of dural carotid-cavernous sinus fistulas in 141 patients. Neuroradiology.

[28] Kobkitsuksakul C, Jiarakongmun P, Chanthanaphak E, Pongpech S (2015). Radiographic Evaluation and Clinical Implications of Venous Connections Between Dural Arteriovenous Fistula of the Cavernous Sinus and Cerebellum and the Pontomedullary Venous System. World Neurosurg.

[29] Cho YD, Rhim JK, Yoo DH, Kang HS, Kim JE, Cho WS (2016). Transvenous microguidewire looping technique for breach of ipsilateral inferior petrosal sinus occlusions en route to cavernous sinus dural arteriovenous fistulas. Interv Neuroradiol.

[30] Kim DJ, Kim DI, Suh SH, Kim J, Lee SK, Kim EY (2006). Results of transvenous embolization of cavernous dural arteriovenous fistula: a single-center experience with emphasis on complications and management. AJNR Am J Neuroradiol.

[31] Tanioka S, Sato Y, Nampei M, Tsuda K, Niwa S, Suzuki H (2016). Cavernous Sinus Dural Arteriovenous Fistula Presenting with Intracerebral Hemorrhage Associated with Chronological Angiographic Demonstration of Disturbed Leptomeningeal Venous Drainage as the Sole Outflow Route. World Neurosurg.

[32] Tong D, Chen X, Lv X, Li K, Xu K, Yu J (2019). Current status of endovascular treatment for dural arteriovenous fistulae in the tentorial middle region: a literature review. Acta Neurol Belg.

[33] Yang HC, Lin CJ, Luo CB, Lee CC, Wu HM, Guo WY (2019). Treatment Outcomes of Cavernous Sinus Dural Arteriovenous Fistulas: Comparison of Radiosurgery and Endovascular Embolisation. Clin Neuroradiol.

[34] Li G, Zhang Y, Zhao J, Zhu X, Yu J, Hou K (2019). Isolated subdural hematoma secondary to Dural arteriovenous fistula: a case report and literature review. BMC Neurol.

[35] Kuwayama N, Endo S, Kitabayashi M, Nishijima M, Takaku A (1998). Surgical transvenous embolization of a cortically draining carotid cavernous fistula via a vein of the sylvian fissure. AJNR Am J Neuroradiol.

[36] Hurley MC, Rahme RJ, Fishman AJ, Batjer HH, Bendok BR (2011). Combined surgical and endovascular access of the superficial middle cerebral vein to occlude a high-grade cavernous dural arteriovenous fistula: case report. Neurosurgery.

[37] Choi JH, Jo KI, Kim KH, Jeon P, Yeon JY, Kim JS (2015). Spontaneous angiographic changes in venous drainage patterns related to symptom changes in patients with untreated cavernous sinus dural arteriovenous fistula. Neuroradiology.

[38] Suh DC, Lee JH, Kim SJ, Chung SJ, Choi CG, Kim HJ (2005). New concept in cavernous sinus dural arteriovenous fistula: correlation with presenting symptom and venous drainage patterns. Stroke.

[39] Thomas AJ, Chua M, Fusco M, Ogilvy CS, Tubbs RS, Harrigan MR (2015). Proposal of Venous Drainage-Based Classification System for Carotid Cavernous Fistulae With Validity Assessment in a Multicenter Cohort. Neurosurgery.

[40] Wenderoth J (2017). Proposal for an improved classification system for cavernous sinus dural arteriovenous fistula (CS-DAVF). J Neurointerv Surg.

[41] Lee SH, Cho WS, Kang HS, Kim JE, Cho YD, Yoo DH (2019). Newly occurring cranial nerve palsy after endovascular treatment of cavernous sinus dural arteriovenous fistulas. J Neurointerv Surg.

[42] Leone G, Renieri L, Enriquez-Marulanda A, Dmytriw AA, Nappini S, Laiso A (2019). Carotid Cavernous Fistulas and Dural Arteriovenous Fistulas of the Cavernous Sinus: Validation of a New Classification According to Venous Drainage. World Neurosurg.

[43] Luo CB, Chang FC, Teng MM, Guo WY, Ting TW (2015). Transvenous embolization of cavernous sinus dural arteriovenous fistula via angiographic occlusive inferior petrous sinus. J Chin Med Assoc.

[44] Wen J, Duan CZ, Huang LJ, Zhang X, He XY, Li XF (2015). Transarterial Onyx Embolization for Patients with Cavernous Sinus Dural Arteriovenous Fistulas Who Have Failed Transvenous Embolization. Cell Biochem Biophys.

[45] Satow T, Murao K, Matsushige T, Fukuda K, Miyamoto S, Iihara K (2013). Superselective shunt occlusion for the treatment of cavernous sinus dural arteriovenous fistulae. Neurosurgery.

[46] Li ZR, Jiang ZB, Huang MS, Zhu KS, Wang Q, Shan H (2010). Transvenous embolization of cavernous sinus dural arteriovenous fistulas using detachable coils and Glubran 2 acrylic glue via the inferior petrosal sinus approach. Eur Radiol.

[47] Carlson AP, Taylor CL, Yonas H (2007). Treatment of dural arteriovenous fistula using ethylene vinyl alcohol (onyx) arterial embolization as the primary modality: short-term results. J Neurosurg.

[48] Yoshida K, Melake M, Oishi H, Yamamoto M, Arai H (2010). Transvenous embolization of dural carotid cavernous fistulas: a series of 44 consecutive patients. AJNR Am J Neuroradiol.

[49] Nossek E, Lombardo K, Schneider JR, Kwan K, Chalif DJ, Setton A (2019). Unilateral Venous Approach to Contralateral or Bilateral Carotid Cavernous Shunts. World Neurosurg.

[50] Jia ZY, Song YS, Sheen JJ, Kim JG, Lee DH, Suh DC (2018). Cannulation of Occluded Inferior Petrosal Sinuses for the Transvenous Embolization of Cavernous Sinus Dural Arteriovenous Fistulas: Usefulness of a Frontier-Wire Probing Technique. AJNR Am J Neuroradiol.

[51] Zhang L, Zeng F, Wang J, Chen F (2019). Finding the Inferior Petrosal Sinus for Embolizing Cavernous Dural Arteriovenous Fistula Using Preoperative Computed Tomography Angiography. World Neurosurg.

[52] Benndorf G, Bender A, Lehmann R, Lanksch W (2000). Transvenous occlusion of dural cavernous sinus fistulas through the thrombosed inferior petrosal sinus: report of four cases and review of the literature. Surg Neurol.

[53] Luo CB, Chang FC, Wang AG, Lin CJ, Guo WY, Ting TW (2016). Transvenous Coil Embolization of Cavernous Sinus Dural Arteriovenous Fistula on a Revised Classification. World Neurosurg.

[54] Mounayer C, Piotin M, Spelle L, Moret J (2002). Superior petrosal sinus catheterization for transvenous embolization of a dural carotid cavernous sinus fistula. AJNR Am J Neuroradiol.

[55] Dabus G, Batjer HH, Hurley MC, Nimmagadda A, Russell EJ (2012). Endovascular treatment of a bilateral dural carotid-cavernous fistula using an unusual unilateral approach through the basilar plexus. World Neurosurg.

[56] Choi JH, Shin YS, Kim BS (2019). Making Microguidewire Loop Facilitates Navigation Through Tortuous or Abruptly Angulated Head and Neck Veins to Access Cavernous Sinus Dural Arteriovenous Fistulas. World Neurosurg.

[57] San Millan Ruiz D, Oka M, Fasel JH, Clatterbuck R, Gailloud P, Murphy K (2007). Transvenous embolization of a dural arteriovenous fistula of the laterocavernous sinus through the pterygoid plexus. Neuroradiology.

[58] Heran MKS, Volders D, Haw C, Shewchuk JR (2019). Imaging-Guided Superior Ophthalmic Vein Access for Embolization of Dural Carotid Cavernous Fistulas: Report of 20 Cases and Review of the Literature. AJNR Am J Neuroradiol.

[59] Fu ZY, Feng Y, Ma C, Chen JC, Krings T, Zhao WY (2019). Endovascular Treatment of Cavernous Sinus Dural Arteriovenous Fistulas via Direct Transorbital Puncture Using Cone-Beam Computed Tomography Image Guidance: Report of 3 Cases. World Neurosurg.

[60] Dashti SR, Fiorella D, Spetzler RF, Albuquerque FC, McDougall CG (2011). Transorbital endovascular embolization of dural carotid-cavernous fistula: access to cavernous sinus through direct puncture: case examples and technical report. Neurosurgery.

[61] Serratrice N, Baucher G, Reyre A, Brunel H, Fuentes S, Dufour H (2019). Management of two cavernous sinus dural arteriovenous fistulae by direct microsurgical approach and catheterization of the superior ophthalmic vein. Neurochirurgie.

[62] Gomez-Paz S, Vergara-Garcia D, Robinson M, Kicielinski KP, Thomas AJ, Ogilvy CS (2019). Coil Embolization of a Carotid-Cavernous Fistula Through Superior Ophthalmic Venous Access via External Jugular Vein Puncture Approach. World Neurosurg.

[63] Alexandre AM, Visconti E, Lozupone E, D'Argento F, Pedicelli A (2017). Embolization of Dural Arteriovenous Fistula of the Cavernous Sinus Through Percutaneous Ultrasound-Guided Puncture of the Facial Vein. World Neurosurg.

[64] Kim MJ, Shin YS, Ihn YK, Kim BM, Yoon PH, Oh SY (2013). Transvenous Embolization of Cavernous and Paracavernous Dural Arteriovenous Fistula through the Facial Vein: Report of 12 Cases. Neurointervention.

[65] Venturi C, Bracco S, Cerase A, Gennari P, Lore F, Polito E (2003). Endovascular treatment of a cavernous sinus dural arteriovenous fistula by transvenous embolisation through the superior ophthalmic vein via cannulation of a frontal vein. Neuroradiology.

[66] Kurata A, Suzuki S, Iwamoto K, Miyazaki T, Inukai M, Abe K (2009). Direct-puncture approach to the extraconal portion of the superior ophthalmic vein for carotid cavernous fistulae. Neuroradiology.

[67] Luo CB, Chang FC, Teng MM, Lin CJ, Wang AG, Ting TW (2016). Aggressive cavernous sinus dural arteriovenous fistula: Angioarchitecture analysis and embolization by various approaches. J Chin Med Assoc.

[68] Chaudhary N, Lownie SP, Bussiere M, Pelz DM, Nicolle D (2012). Transcortical venous approach for direct embolization of a cavernous sinus dural arteriovenous fistula: technical case report. Neurosurgery.

[69] Fioravanti A, Fiaschi P, Badaloni F, Calbucci F, Leonardi M (2019). Transcranial approach for surgical-combined-endovascular treatment of a cavernous dural arteriovenous fistula: the superficial sylvian vein route. J Neurosurg Sci.

[70] Akamatsu Y, Sato K, Endo H, Matsumoto Y, Tominaga T (2017). Single-Session Hematoma Removal and Transcranial Coil Embolization for a Cavernous Sinus Dural Arteriovenous Fistula: A Technical Case Report. World Neurosurg.

[71] Cabral De Andrade G, Alves HP, Parente R, Salvarani CP, Climaco VM, Pereira ER (2012). Spontaneous isolated dural arteriovenous fistula of the cavernous sinus: endovascular approach via the foramen ovale. A technical note. Interv Neuroradiol.

[72] Gil A, Lopez-Ibor L, Lopez-Flores G, Cuellar H, Murias E, Rodriguez-Boto G (2013). Treatment of a carotid cavernous fistula via direct transovale cavernous sinus puncture. J Neurosurg.

[73] Nelson PK, Russell SM, Woo HH, Alastra AJ, Vidovich DV (2003). Use of a wedged microcatheter for curative transarterial embolization of complex intracranial dural arteriovenous fistulas: indications, endovascular technique, and outcome in 21 patients. J Neurosurg.

[74] Yu J, Guo Y, Xu B, Xu K (2016). Clinical importance of the middle meningeal artery: A review of the literature. Int J Med Sci.

[75] Ashour R, Chavali R (2015). Neuromeningeal access for transarterial intravenous carotid-cavernous fistula embolization. Interv Neuroradiol.

[76] Hou K, Ji T, Guo Y, Xu B, Xu K, Yu J (2019). Current Status of Endovascular Treatment for Dural Arteriovenous Fistulas in the Superior Sagittal Sinus Region: A Systematic Review of the Literature. World Neurosurg.

[77] Geibprasert S, Pongpech S, Armstrong D, Krings T (2009). Dangerous extracranial-intracranial anastomoses and supply to the cranial nerves: vessels the neurointerventionalist needs to know. AJNR Am J Neuroradiol.

[78] Hung YC, Mohammed N, Kearns KN, Chen CJ, Starke RM, Kano H (2019). Stereotactic Radiosurgery for Cavernous Sinus Versus Noncavernous Sinus Dural Arteriovenous Fistulas: Outcomes and Outcome Predictors. Neurosurgery.

[79] Park SH, Park KS, Kang DH, Hwang JH, Hwang SK (2017). Stereotactic Radiosurgery for Dural Carotid Cavernous Sinus Fistulas. World Neurosurg.

[80] Wu CA, Yang HC, Hu YS, Wu HM, Lin CJ, Luo CB (2019). Venous outflow restriction as a predictor of cavernous sinus dural arteriovenous fistula obliteration after Gamma Knife surgery. J Neurosurg.

[81] Jung KH, Kwon BJ, Chu K, Noh Y, Lee ST, Cho YD (2011). Clinical and angiographic factors related to the prognosis of cavernous sinus dural arteriovenous fistula. Neuroradiology.

[82] Zhang J, Lv X, Jiang C, Li Y, Yang X, Wu Z (2010). Transarterial and transvenous embolization for cavernous sinus dural arteriovenous fistulae. Interv Neuroradiol.

[83] de Castro-Afonso LH, Trivelato FP, Rezende MT, Ulhoa AC, Nakiri GS, Monsignore LM (2018). Transvenous embolization of dural carotid cavernous fistulas: the role of liquid embolic agents in association with coils on patient outcomes. J Neurointerv Surg.

[84] Nishimuta Y, Awa R, Sugata S, Nagayama T, Makiuchi T, Tomosugi T (2017). Long-term outcome after endovascular treatment of cavernous sinus dural arteriovenous fistula and a literature review. Acta Neurochir (Wien).

[85] Castro-Afonso LH, Trivelato FP, Rezende MT, Ulhoa AC, Nakiri GS, Monsignore LM (2019). The routes for embolization of dural carotid cavernous fistulas when the endovascular approach is indicated as a first-line strategy. Interv Neuroradiol.

[86] Hassan T, Rashad S, Aziz W, Sultan A, Ibrahim T (2015). Endovascular Modalities for the Treatment of Cavernous Sinus Arteriovenous Fistulas: A Single-Center Experience. J Stroke Cerebrovasc Dis.

[87] Klisch J, Huppertz HJ, Spetzger U, Hetzel A, Seeger W, Schumacher M (2003). Transvenous treatment of carotid cavernous and dural arteriovenous fistulae: results for 31 patients and review of the literature. Neurosurgery.

[88] Trivelato FP, Manzato LB, Filho PMM, Ulhoa AC, Vanzin JR, Abud DG (2018). Transorbital Cavernous Sinus Direct Puncture: Alternative to treat dural arteriovenous fistula. Clin Neuroradiol.

[89] Chan HH, Asadi H, Dowling R, Hardy TG, Mitchell PJ (2014). Facial nerve injury as a complication of endovascular treatment for cavernous dural arteriovenous fistula. Orbit.

[90] Kohta M, Fujita A, Tanaka J, Sasayama T, Hosoda K, Kohmura E (2018). Novel Segmentation of Placed Coils in the Treatment of Cavernous Sinus Dural Arteriovenous Fistulas Provides a Reliable Predictor of the Long-Term Outcome in Abducens Nerve Palsy. World Neurosurg.

[91] Kashiwazaki D, Kuwayama N, Akioka N, Kuroda S (2014). Delayed abducens nerve palsy after transvenous coil embolization for cavernous sinus dural arteriovenous fistulae. Acta Neurochir (Wien).

[92] Nicholson P, Hilditch C, Brinjikji W, Krings T (2019). Asystole during onyx embolisation of a dural AV fistula: The trigeminocardiac reflex. Interv Neuroradiol.

[93] Amiridze N, Zoarski G, Darwish R, Obuchowski A, Solovevchic N (2009). Embolization of a Cavernous Sinus Dural Arteriovenous Fistula with Onyx via Direct Puncture of the Cavernous Sinus through the Superior Orbital Fissure: Asystole Resulting from the Trigeminocardiac Reflex. A Case Report. Interv Neuroradiol.

[94] Xianli L, Chuhan J, Youxiang L, Aihua L, Ming L, Peng J (2007). Trigeminocardiac reflex in transvenous packing of the cavernous sinus with onyx for dural arteriovenous fistula. A case report. Neuroradiol J.

[95] Lv X, Li Y, Jiang C, Wu Z (2010). The incidence of trigeminocardiac reflex in endovascular treatment of dural arteriovenous fistula with onyx. Interv Neuroradiol.

[96] Kai Y, Hamada JI, Morioka M, Yano S, Ushio Y (2004). Brain stem venous congestion due to dural arteriovenous fistulas of the cavernous sinus. Acta Neurochir (Wien).

